# *Acinetobacter* spp. in neonatal sepsis: an urgent global threat

**DOI:** 10.3389/frabi.2024.1448071

**Published:** 2024-09-03

**Authors:** Kamla Pillay, Anirban Ray-Chaudhuri, Seamus O’Brien, Paul Heath, Mike Sharland

**Affiliations:** ^1^ Institute of Infection & Immunity, St George’s, University of London, London, United Kingdom; ^2^ Neonatal Unit, University College Hospital (UCH), London, United Kingdom; ^3^ Critical Care, Whittington Hospital, London, United Kingdom; ^4^ Research & Development, Global Antibiotics Research and Development Partnership, Geneva, Switzerland

**Keywords:** *Acinetobacter* spp., *Acinetobacter baumanii*, neonatal sepsis, biofilm, antibiotic resistance, virulence traits, natural transformation

## Abstract

Neonatal sepsis causes substantial morbidity and mortality, the burden of which is carried by low-income countries (LICs). The emergence of multidrug-resistant pathogens in vulnerable neonatal populations poses an urgent threat to infant survival. *Acinetobacter* spp. are increasingly responsible for severe disease in neonates globally. The cause of this escalation remains unclear, but host, pathogen and environmental factors are all likely to contribute. *Acinetobacter* spp. strains are frequently resistant to the first line empirical treatment for neonatal sepsis as recommended by the World Health Organization (WHO), ampicillin and gentamicin, rendering these antibiotics ineffectual in many critically ill neonates. The resultant escalation to broader spectrum antibiotic regimens in neonatal intensive care units (NICUs) worldwide has led to the emergence of more resistant strains, including carbapenem-resistant *Acinetobacter baumanii* (CRAB), resulting in infections that are ever more difficult to treat. While some existing antimicrobial agents are under consideration for treatment of *Acinetobacter* spp. infections, the majority remain a long way from clinical use in neonates. Further research into the clinical phenotype of these infections, transmission dynamics and preventative measures are urgently needed to reduce neonatal deaths. This review aims to summarise the role of *Acinetobacter* spp. in neonatal sepsis, including host, pathogen and environmental factors, the global epidemiology and clinical features of the disease, the treatment options, and future research priorities.

## Introduction

Neonatal sepsis, defined as a severe systemic infection occurring in newborn infants within the first 28 days of life, contributes substantially to neonatal mortality and morbidity worldwide (Fleischmann et al., 2021). The non-specific clinical features coupled with limited diagnostic test accuracy make clinical diagnosis of the condition challenging (Shane et al., 2017). Clinically the disease is often divided into early-onset sepsis (EOS), characterised by a positive blood or cerebrospinal fluid culture within the first 72 hours of life, and late-onset sepsis (LOS), which manifests after this initial period (Li et al., 2020; Sands et al., 2021). While EOS and LOS are traditionally thought to have different causative pathogens, a recent global observational cohort study found that all commonly identified pathogens were found in both EOS and LOS, with relatively minor differences in proportion (Russell et al., 2023). Risk factors for neonatal sepsis are primarily related to risk of pathogen exposure (e.g. maternal colonisation, prolonged rupture of membranes and NICU admission) and relative immaturity of immune system development (e.g. prematurity) (Shane et al., 2017).

In many cases of neonatal sepsis a causative organism is not definitively found, attributed in part to poor diagnostic test accuracy (Klingenberg et al., 2018). Blood cultures are seen as the ‘gold standard’ diagnostic test, however many factors reduce their diagnostic accuracy including the small volume of blood from neonatal samples, maternal antibiotic administration prior to delivery, and laboratory methodology (Camacho-Gonzalez et al., 2013). Causative pathogen distribution varies based on setting, with a predominance of Gram-negative pathogens worldwide, though with more Gram-positive pathogens (primarily *Staphylococcal* spp. and *Streptococcal* spp.) in higher income settings (Fleischmann et al., 2021). Longitudinal data from one South African neonatal unit show increasing Gram-negative prevalence over a 10-year period with static or falling rates of their Gram-positive counterparts (Thomas et al., 2024). *Acinetobacter* spp., primarily *Acinetobacter baumanii*, are increasingly implicated in the neonatal population, outpacing comparable Gram-negative pathogens such as *P. aeruginosa*. This review aims to propose some possible mechanisms for this increase, with consideration of microbial, host and environmental factors, to summarise clinical neonatal data, and to discuss current and novel treatment options, preventative measures and future research priorities.

## The host

The transition from an intrauterine to an extrauterine environment results in sudden exposure of neonates to environmental microbes. Microbial colonisation in the postnatal period is key to development of a healthy microbiome, with most ‘good’ bacteria originating from maternal flora and breast milk. The early microbiome contributes to immune system maturation (Sanidad and Zeng, 2020). However, this is a vulnerable time for colonisation with potentially harmful pathogens, given the relative absence of ‘good’ bacteria in the newborn’s gut and skin, particularly in neonates in nosocomial settings (Hartz et al., 2015). The neonatal gut is immature, with low levels of acid secretion, protective mucus and motility, resulting in an increased risk of bacterial translocation through the gut wall (Basu, 2015). Colonisation with Gram-negative bacteria is thought to increase the risk of invasive infection in neonates, though further evidence for this is needed for *Acinetobacter* spp. infection (Folgori et al., 2018). One study found the presence of Gram-negative bacteria in gastric aspirates and stool samples of hospitalised neonates significantly increased the chance of invasive infection with that same strain. Interestingly, of the neonates with invasive isolates, only 20% of those with *A. baumanii* isolated in blood cultures had previously been colonised with the same strain. For comparison, of neonates with *Klebsiella pneumoniae* isolated from their blood culture, 90% had been previously colonised with this same strain. This suggests that while gut colonisation may be linked to invasive *Acinetobacter* spp. infection, other sources of acquisition, without colonisation, may play a larger role compared to other common Gram-negative pathogens, though it is unclear if culture methods used may have been less appropriate for *Acinetobacter* spp. (Das et al., 2011). Additional studies with larger sample sizes are required to better characterise this association.

Multiple factors render neonates uniquely susceptible to infections. Neonates lack acquired immunity, resulting in a dependence on innate immune mechanisms, though the lack of a functional complement system means that even these responses are dampened, particularly in premature infants (McGreal et al., 2012; Hensler et al., 2022). Neonates are thought to have impaired neutrophil function, possibly due to a lower proportion of mature neutrophils when compared to older children and adults (Makoni et al., 2016). While the interactions between *Acinetobacter* spp. and the neonatal immune system are not well described, in adult populations neutropenic patients are particularly at risk, even when compared to other immunocompromised individuals (Karim et al., 1991; Chen, 2020). Immature neutrophil function may therefore contribute to neonatal vulnerability to *Acinetobacter* spp. infection, though the mechanisms behind this require exploration.

## The pathogen

*Acinetobacter* spp. are Gram-negative coccobacilli with emerging importance in neonatal infections. The *Acinetobacter calcoaceticus-baumannii* (Acb) complex is a group of phenotypically indistinguishable pathogens including *Acinetobacter baumannii*, *Acinetobacter pitii* and *Acinetobacter nosocomialis*, among others. While there are reports of both Acb and non-Acb *Acinetobacter* spp. as causative organisms for neonatal sepsis, *Acinetobacter baumannii* is of particular clinical importance. For the purposes of this review, *Acinetobacter* spp. is used to encompass both Acb and non-Acb pathogens.

*A. baumanii* is one of six ESKAPE pathogens, named for their ability to escape the effects of antimicrobial drugs. These pathogens are the leading cause of nosocomial infections worldwide, and have been named high priority by the WHO. *A. baumannii* was designated in ‘critical’ need for novel antibiotic development (WHO, 2017).

The microbiological characteristics of *Acinetobacter* spp. partially explain its emergence as a neonatal pathogen. Firstly, *Acinetobacter* spp. are unique in the diversity of their ecological niche when compared to other pathogenic bacteria. In the natural world they are found in soil, rivers, animals and humans, reflecting the range of environments in which they thrive. One enabler of this is their resistance to external stresses including desiccation, which is rare among Gram-negative bacteria and confers a substantial survival advantage particularly in nosocomial settings (Jawad et al., 1998; Mea et al., 2021). Some isolates have been reported to remain viable for up to 3 months on dry surfaces (Wendt et al., 1997; Jawad et al., 1998). Though the mechanisms behind this tolerance are unclear, biofilm formation, in which capsular polysaccharides envelop the bacterial cells, is likely to contribute to the maintenance of bacterial cell hydration despite adverse external conditions (Espinal et al., 2012). In addition to desiccation resistance, *Acinetobacter* spp. also demonstrate some resistance to disinfectants, mediated by multidrug efflux pumps. Lanjri et al. (2017) found that from a range of clinical and environmental *A.baumanii* isolates, while all were susceptible to antiseptics and disinfectants in their purest form, clinical isolates required a higher concentration to achieve sterility than their environmental counterparts. These factors give *Acinetobacter* spp. an unparalleled ability to colonise nosocomial settings.

Secondly, *Acinetobacter* spp. have remarkable intrinsic and acquired resistance to antibiotics. Two key intrinsic β-lactamases, ABC and OXA-51, confer some resistance to penicillins, cephalosporins, and carbapenems (Bonomo and Szabo, 2006; Zavascki et al., 2010). However, without additional acquired resistance mechanisms they remain relatively susceptible. For example, strains described in the 1970s were successfully treated with agents such as ampicillin, chloramphenicol or gentamicin (Vázquez-López et al., 2020). The more recent emergence of multidrug resistant strains can be attributed to exceptional genetic plasticity, enabling *Acinetobacter* spp. to rapidly give and receive antibiotic resistance genes (ARGs) via horizontal gene transfer (Da Silva and Domingues, 2016). Carbepenem-resistant *Acinetobacter Baumanii* (CRAB) first emerged in the 1990s, primarily in adult intensive care settings, and has since emerged in NICUs worldwide. OXA23 is the most common carbapenem resistance gene, making up 75% of plasmid-acquired OXAs in *Acinetobacter* spp., though OXA 40, 153 and 235 are also frequently identified (Vázquez-López et al., 2020). One study comparing 20 environmental and clinical isolates of *A.baumanii* found the clinical isolates to demonstrate significantly higher antibiotic resistance than the environmental isolates, with 12/13 (92%) of clinical isolates characterised as extensively drug resistant (XDR), compared to 1/7 (14%) of environmental isolates (Havenga et al., 2022).

Finally, *Acinetobacter* spp. have a number of virulence factors that enable pathogen success at every stage of pathogenesis. These include outer membrane proteins (Omps) to facilitate cell membrane attachment and a Type VI secretion system to kill competitor bacterial cells (Shadan et al., 2023). The plastic genome supports transfer of virulence genes from bacteria to bacteria, enabling rapid evolution in response to changing environments and stressors.

## The environment

The NICU environment contributes significantly to neonatal infection risk. First, it confers a high risk of exposure to multidrug resistant bacteria. Overcrowded units in under-resourced settings are at particular risk, often lacking the space, capacity or funds to institute regular, effective disinfection of surfaces or equipment. Invasive procedures, such as central vascular access and endotracheal intubation, further increase risk of exposure given their penetration of natural immune barriers such as the skin. Some NICUs are forced to care for multiple patients in single incubators or cots, further increasing risk of bacterial transmission between patients. Many neonatal pathogens thrive in one environmental niche within a NICU setting, for example *Pseudomonas aeruginosa* is associated with water reservoirs, and has therefore been frequently identified in humidified ventilator tubing and taps in neonatal units (Jefferies et al., 2012). The unique adaptability of *Acinetobacter* spp. enables it to occupy multiple different environmental niches within the neonatal unit, including both moist and dry environments, which may explain its growth in these settings. Reports of *Acinetobacter* spp. colonisation on neonatal units include detection from wound and device dressings, sinks and dry medical equipment such as syringe drivers (Tsiatsiou et al., 2015; Woon et al., 2023). The lack of a singular environmental niche complicates the design of control interventions aimed at preventing NICU-based transmission of *Acinetobacter* spp.

## The disease: global epidemiology and prevalence

A PubMed search was performed to identify systematic reviews and meta-analyses reporting on causative organisms of neonatal sepsis globally. **Table 1** lists the identified systematic reviews that reported *Acinetobacter* spp. as an invasive neonatal isolate. In the six publications identified *Acinetobacter* spp. infections were reported to comprise an overall proportion 1-6% of all cases of culture-positive neonatal sepsis included in the analysis. The highest overall prevalence was reported in an analysis of 20,828 positive cultures from low and lower-middle income countries globally (Wen et al., 2021). Okomo et al., who collated data from 151 Sub-Saharan African countries, reported that Central African countries were found to have the highest prevalence (11%), while West African countries had the lowest (2%). Regional variation is marked and Wen et al. concluded that the prevalence of invasive *Acinetobacter* spp. neonatal infections is higher in Asia than in Africa, a finding which is reflected by DENIS (2016), which reported a 22% *Acinetobacter* spp. prevalence in culture-positive neonatal sepsis among multiple sites in Delhi, India DENIS, 2016. A number of regional studies, for example in Cuba, China and Nigeria, found no *Acinetobacter* spp. in neonatal isolates, further reflecting the uneven global distribution (Medugu et al., 2018; Oliva et al., 2021). While the underlying causes for this global distribution remain unclear, regional antibiotic prescribing practices may provide a positive selective pressure. Unit to unit variation is noted even between units in similar geographical regions, supporting the hypothesis that unit-level colonisation may drive infections in the neonatal population (Sands et al., 2021; Russell et al., 2023).

**Table 1:** Systematic review and meta-analyses including *Acinetobacter* spp. proportion among culture-positive cohorts of neonates with sepsis.

Systematic reviews and meta-analysis reporting *Acinetobacter* spp. prevalence							
Study name	Region	Inclusion criteria	Number of papers	Number of neonates/isolates	Isolate source	Prevalence of *Acinetobacter* spp. among positive cultures (%, 95% CI)	Acinetobacter additional subanalysis (if done) e.g. regional distribution
Okomo et al (Okomo et al., 2019)	Sub-Saharan Africa	1980-2018 Infectious aetiology identified	151	84534 neonates 31564 blood cultures taken, of which 7856 positive and 1738 LPs performed of which 135 positive	Blood and CSF	Pooled 1980 - 2007: for sepsis: 5%, 2-7 for meningitis*: 4, 1-8 2008 - 2018 for sepsis: 5, 3-7 for meningitis*: 10, 4-17	Regional prevalences: Central Africa 11%; Eastern 5%; Southern 7%; West 2%
Review (Wen et al., 2021)	19 low and lower middle income countries (according to World Bank 2019)	2010-2021 Infectious ethology identified	88	20,828 positive blood/CSF cultures. Number of neonates not documented	Blood and CSF	Pooled 6, 4-10	More common in Asia than Africa 84% 3CG resistance 70% gentamicin 64% amikacin 71% ciprofloxacin 42% carbapenem resistance estimated
Li et al (Li et al., 2018)	China	2009-2014 NICU patients Infectious aetiology identified	71	8080 positive blood cultures	Blood only	Pooled 1.6, 1.3-2	nil
(Akbarian-Rad et al., 2020)	Iran	2000-2019 Infectious ethology identified	22	14683 neonates	Blood only	Pooled 4.61, 2.17-7.74	nil
(Khalil et al., 2020)	Middle East	2000-2017 Early onset sepsis (Susceptibility data includes late onset sepsis)	33	2215 isolates	Blood only	Total 55/2215 (2%)	No significant difference in prevalence in high and middle income countries
(Medugu et al., 2018)	Nigeria	1987 - 2017 Infectious aetiology identified	24	7802 suspected cases 2280 positive blood culture	Blood only	Total 0/2280 (0%)	Possible sampling issue - 89% of samples processed by manual culture rather than automated, authors suggest may reduce sensitivity and range of detection

*numbers given for cases defined as meningitis in meta-analysis, however criteria for meningitis case definition not specified.

NS, not stated.

## The disease: risk factors and clinical phenotype

There is limited published evidence on risk factors and clinical features of *Acinetobacter* spp. infection in a neonatal population, compared to other causative organisms. One global meta-analysis on risk factors for carbapenem and polymixin-resistant infections in neonates found that intubation, prematurity, low birth weight and mechanical ventilation were common risk factors for these infections in almost all countries (Osei Sekyere et al., 2021). Other studies report central venous lines and previous antibiotic use as additional risk factors (Al Jarousha et al., 2009; Zarrilli et al., 2012; Maciel et al., 2018; Ulu-Kilic et al., 2018). No large-scale studies were identified that define a clinical phenotype for neonatal *Acinetobacter* spp. infections.

There are a number of reports suggesting that *Acinetobacter* spp. is overrepresented in cases of neonatal meningitis, which is associated with high mortality and adverse neurodevelopmental outcomes. Interestingly, Okomo et al., who analysed a total of 7856 positive cultures from Sub-Saharan Africa, found that *Acinetobacter* spp. made up 5% of isolates from blood cultures and 10% from CSF cultures. A similar proportion is reported in a multisite South African study where *A. baumanii* was isolated in 13% and 23% of positive blood and CSF cultures, respectively (Mashau et al., 2022). It remains to be determined whether this reflects a preferential tropism for CSF with *Acinetobacter* spp., or disease progression secondary to antimicrobial resistance and first-line treatment failure.

## The disease: mortality

Mortality of neonatal sepsis secondary to *Acinetobacter* spp. infection is very high. NeoObs, a global observational trials of neonatal sepsis reported a mortality of approximately 30% in neonates with culture-positive sepsis secondary to *Acinetobacter* spp. (Russell et al., 2023). For comparison, mortality secondary to any Gram-negative isolate was reported as 21% in NeoObs. These high estimates are reflected in outbreak mortality, with the case fatality rate in NICU outbreaks caused by *A. baumanii* estimated to be 34% (Birt et al., 2016). Possible reasons for high *Acinetobacter* spp. mortality includes higher intrinsic disease severity and AMR-related treatment failure. Individual regions report even higher mortality, for example a multi-site study in India found a 59% mortality from *Acinetobacter* spp. culture-positive sepsis, compared to mortality of 48% for all other isolates (DENIS, 2016). Regional differences in mortality may be explained by quality of supportive care or virulence factors in local *Acinetobacter* spp. strains.

## Antibiotic resistance and virulence factors in neonatal isolates

Molecular characterisation of invasive neonatal *Acinetobacter* spp. may bring clues to the host-pathogen dynamics in this vulnerable population. However, there is currently a lack of published data comparing virulence genes in invasive neonatal isolates when compared with environmental and/or invasive isolates in other populations. A number of virulence factors have been reported from neonatal isolates, though no meta-analysis was found for these, with most focusing on antibiotic resistance genes rather than virulence.

Wen et al. reported 70%, 84% and 42% resistance of *Acinetobacter* spp. isolates to gentamicin, third generation cephalosporin and carbapenems, respectively. These numbers are of clinical relevance in light of the WHO recommendations to use ampicillin and gentamicin first-line and ceftriaxone second line in the empirical treatment of neonatal sepsis worldwide. These high resistance rates are also reflected in recent cohort studies (see **Table 2**), and demonstrate the growing burden of CRAB worldwide. The most commonly mentioned ARGs are in the OXA group, particularly OXA23 which confers carbapenem resistance.

**Table 2:** Global cohort studies recruiting neonates with sepsis reporting *Acinetobacter* spp. proportions.

Study name	Countries	Age	Inclusion criteria	Number of participants/isolates	Number with culture positive sepsis	*Acinetobacter* spp. proportion	*Acinetobacter* spp. mortality	*Acinetobacter* spp. identification	*Acinetobacter* spp. resistance
Global and/or multiple site cohort studies									
NeoObs (Russell et al., 2023)	Bangladesh, China, India, Thailand, Vietnam, Kenya, South Africa, Uganda, Italy, Greece, Brazil	0-60 days	clinical features and laboratory results	3195 cases	564/3195 (18%)	72/564 (13%)	24/72 (33%)	No species breakdown	*Acinetobacter* spp.: meropenem resistance 50/70 (70%) gentamicin resistance 50/70 (70%) amikacin resistance 51/70 (72%)
BARNARDS (Sands et al., 2021)	Bangladesh, Ethiopia, India, Nigeria, Pakistan, Rwanda, and South Africa	0-60 days	clinical features and risk factors	36285 cases	2483/36285 (7%)	49/2483 (2%)	*A.baumanii* 5/38 (13%) Non-baumanii *Acinetobacter* spp. 0/11 (0%)	*A.baumanii* 38/49 (78%) A. nosocomialis 3/49 (6%) Non-Acb 6/49 (12%) Undifferentiated 2/49 (4%)	*Acinetobacter* spp.: Meropenem resistance 23/49 (47%) Amikacin 24/49 (49%) Tigecycline 1/49 (2%)
(Mashau et al., 2022)	South Africa (256 healthcare facilities)	0-27 days	Clinical features	37631 cases 43438 positive isolates (blood and CSF)	43438	5686/43438 (13%) present in 13% of BC isolates but in 23% of CSF isolates		No species breakdown	90% of *A baumanii* MDR

## Current and future treatment options

CRAB now dominates the global burden of *Acinetobacter* spp. infections, leaving few available treatment options (Osei Sekyere et al., 2021). Though pre-existing and novel antimicrobial options are being explored, resistance mechanisms to these agents often develop quickly. Polymyxin antibiotics, such as polymyxin B and colistin, seem among the most active agents against *Acinetobacter* infections at present (Kassamali et al., 2015). While these agents are used in some NICUs, their clinical use is hampered by delivery issues, concerns about toxicity and rapidly developing resistance (Cai et al., 2012; Bostanghadiri et al., 2024). Novel tetracyclines, such as tigecycline, have also shown some promise in the treatment of paediatric infections (Sharland et al., 2019). However, these medications have a high toxicity profile, limited clinical data and lack evidence to support a neonatal dose (Falagas et al., 2015). Cefiderocol, a novel cephalosporin antibiotic, has potent *in vitro* activity against a wide range of Gram-negative pathogens, with some evidence to suggest that this includes *Acinetobacter* spp. Surveillance data suggest that most clinical isolates are sensitive to cefiderocol, though the mechanisms of resistance in the remainder are not fully understood and clinical efficacy data is limited (Kollef et al., 2023). There is a lack of data to support a neonatal dose, though a pharmacokinetic study is currently ongoing for infants under 3 months of age (NCT06086626). Durlobactam, a novel β-lactamase inhibitor, combined with sulbactam, which has intrinsic activity against *Acinetobacter* spp., is another possible therapeutic option. A recent Phase 3 trial found this combination non-inferior to colistin in adults, with further trials underway (Kaye et al., 2023). Several landmark clinical trials have compared combination therapies specifically in adults with *A. baumanii* infection, however there are few paediatric equivalents. Drug development and assessment focused on neonates is urgently required to determine an optimal dose, efficacy and safety of novel regimens in this population, particularly given their unique pharmacokinetic profile which rapidly changes with postnatal age (Williams et al., 2022; Poggi and Dani, 2023; Bamford et al., 2024).

## Disease prevention: microbiological surveillance and infection control measures

An obvious strategy to reduce *Acinetobacter* spp. infections in neonates is to target NICU colonisation and transmission. There remains inadequate evidence for the majority of infection prevention and control interventions in this setting. One intervention known to reduce infant colonisation with pathogenic Gram-negative pathogens is intermediate Kangaroo Mother Care (iKMC), which promotes colonisation with non-pathogenic microbiota organisms and reduces neonatal sepsis-related mortality (Arya et al., 2023). A recent Cochrane review assessing the effects of infant isolation or cohorting concluded there was insufficient evidence for these interventions, and that more high quality trials were needed (Hanna et al., 2023). Another meta-analysis concluded that there was insufficient evidence that implementation of surveillance methods reduced NICU-associated infections (Birt et al., 2016). Larger scale studies specifically designed to evaluate infection control interventions including behavioural analysis are required in order to determine optimal *Acinetobacter* spp. control strategies in this setting, particularly given its lack of clear primary environmental niche.

## Disease prevention: vaccination

As conventional antibiotic options dwindle, there is an increasing demand for alternative strategies in the global fight against *A. baumanii* infections, with recent research focusing on vaccine development. The use of reverse vaccinology has identified numerous targets including outer membrane vesicles, outer membrane protein A, auto-transporter, biofilm-associated protein, K1 capsular polysaccharide, and poly-(beta-1,6)-N-acetyl glucosamide. Active vaccinations have been demonstrated to confer protection in animal models, while a monoclonal antibody targeting K1 capsular polysaccharide has also shown protective effects *in vivo* (Russo et al., 2013; Chen, 2015). Both active and passive vaccinations could be helpful for neonates, for example via maternal vaccination or targeting high risk populations, though these may be challenging to identify. While these efforts are promising, at present no *A. baumannii* vaccine candidates have progressed beyond Phase I trials, in part because of the pathogen’s wide antigenic variability (Singh et al., 2022).

## Future research priorities

Given the burgeoning harm caused by *Acinetobacter* spp. in neonates, a renewed interest is required and research priorities need to be established. First, further molecular genotyping is needed to compare neonatal strains of *Acinetobacter* spp. to strains derived from different populations. The use of molecular techniques on invasive isolates (as compared with colonising isolates) could illuminate the pathogen-specific factors that enable neonatal infection. Should an apparent propensity to cause meningitis be consistently shown, a comparison of CSF and blood isolates could help to understand the mechanisms for this. Sample collection would be best performed as a subproject of existing initiatives, such as the African severe neonatal infection platform (SNIP-AFRICA, 2023). As part of this, clinical data could be used to ascertain whether a clinical phenotype for *Acinetobacter* infection exists in neonates with sepsis that differs from sepsis caused by other pathogens. Pre-existing trials, for example the NeoSep1 study (ISRCTN48721236), could be used as a platform for this data collection.

Second, transmission dynamics using environmental sampling and colonisation data must be used to better characterise the most common ecological niches in *Acinetobacter* spp. spread in a NICU setting. *Acinetobacter* spp.-specific infection control interventions should be created and evaluated with robust study designs. Research must prioritise effective and cheap interventions that combat the most frequent ecological niches identified, combined with behavioural change analysis to ensure that these interventions result in sustained results. Finally, there is a need to prioritise development of novel treatments for *Acinetobacter* spp. infections in the neonatal population, which has been underserved in existing antimicrobial development.

## Conclusion

*Acinetobacter* spp. play an ever growing role in neonatal sepsis and meningitis worldwide, with the burden of disease resting in low income settings. Host, pathogen and environmental factors result in increasing numbers of infections with high mortality. Here, we suggest a number of research priorities going forward; microbiological and clinical characterisation of neonatal sepsis secondary to *Acinetobacter* spp. as well as evaluations of infection control interventions and prioritisation of neonatal drug development.
